# Blepharoptosis among Korean adults: age-related prevalence and threshold age for evaluation

**DOI:** 10.1186/s12886-020-01350-y

**Published:** 2020-03-13

**Authors:** Ji-Sun Paik, Kyungdo Han, Suk-Woo Yang, Yongkyu Park, Kyungsun Na, Wonkyung Cho, Su-Kyung Jung, Sungeun Kim

**Affiliations:** 1 Department of Ophthalmology, Yeouido St. Mary’s Hospital, College of Medicine, The Catholic University of Korea, Seoul, South Korea; 2 Department of Biostatistics, The Catholic University of Korea, 222 Banpo-daero, Seocho-Gu, Seoul, 137-701 South Korea; 3 Department of Ophthalmology and Visual Science, Seoul St. Mary’s Hospital, College of Medicine, The Catholic University of Korea, 222 Banpo-daero, Seocho-Gu, Seoul, 137-701 South Korea; 4 Department of Ophthalmology, Daejeon St. Mary’s Hospital, College of Medicine, The Catholic University of Korea, Daejeon, South Korea; 5 Eyeclinc, Center for Clinical Center, National Cancer Center, Goyang-si, South Korea

**Keywords:** Blepharoptosis, Korean population, Age-related prevalence, Threshold age

## Abstract

**Background:**

To evaluate the prevalence of blepharoptosis among Korean adults and the characteristics of blepharoptosis patients, and to determine an appropriate age threshold for recommending blepharoptosis evaluation.

**Methods:**

The Korean National Health and Nutrition Examination Survey (KNHANES-V) was conducted in 2010–2012. We extracted data on 17,878 Korean adults aged more than and equal to 19 years included in KNHANES-V, and determined blepharoptosis prevalence according to age, to determine the cutoff age for recommending blepharoptosis evaluation. We also determined the possible association between blepharoptosis and obesity parameters, such as body mass index (BMI) and waist circumference (WC).

**Results:**

There was astrong association between older age and the prevalence of blepharoptosis. The cutoff age for recommending blepharoptosis evaluation was 63 years for males, 70 years for females, and 66 years for all patients. Patients with a high BMI and large WC had a higher prevalence of blepharoptosis in all age groups except for those aged over 80 years. The association of blepharoptosis with BMI according to age group showed that in the 50–59 and 60–69 years age groups, blepharoptosis prevalence and BMI were higher. However, in the 70–79 and 80–89 years age groups, extremely obese patients (BMI > 30) showed a decreased blepharoptosis prevalence.

**Conclusions:**

Moderate to severe blepharoptosis can result in poor visual function and exacerbate headaches and depression, leading to decreased quality of life. This study proposed an appropriate age threshold for recommending evaluation of patients with blepharoptosis among the general population of Korea.

## Background

Blepharoptosis, which refers to the drooping of one or both eyelids, affects both the function and appearance of the eyes [1]. In aponeurotic blepharoptosis, the levator aponeurosis and the underlying Müller’s muscle are detached from the tarsus, attenuated in functional terms, and elongated due to the aging process or other mechanisms [2–4]. As a result, droopiness of the eyelids may interfere with the superior visual field and, in moderate to severe cases, the central visual field [3, 4]. Blepharoptosis also results in an altered facial appearance, with patients reporting that they looked “tired” and “more aged.” Following ptosis repair surgery, patients ranked eye and eyelid appearance as the most important postoperative changes with respect to their improved visual field [5].

Psychological distress has been reported to be associated with the presence of blepharoptosis. Richards et al. reported that patients with blepharoptosis had higher levels of anxiety, depression, and concerns regarding their appearance than the general population [5]. When questioned about their visual function-related quality of life, Briceño et al. reported that after surgery for ptosis repair, patients reported a significant increase in visual function [6]. Based on these findings, physicians should be concerned about appearance-related distress and compromised psychological well-being among patients with ptosis.

Droopiness of the eyelids can also lead to reflexive contraction of the occipitofrontalis muscle in patients with ptosis. As a result of tonic reflexive contraction of the occipitofrontalis muscle, ptosis patients may also experience tension-related headaches [7–9]. Similarly, Simsek reported that surgery for ptosis and blepharoptosis provided significant relief from tension-related headaches, and resulted in an improved quality of life [10].

Involutional blepharoptosis is a result of the aging process combined with periorbital changes [11], and can precipitate functional [6] and psychological distress [4] and certain types of severe headache [7–9]. All blepharoptosis symptoms can usually be controlled by surgery to remove excessive upper eyelid skin and reinforce the levator apouneurosis [12]. This study is the first to assess the prevalence of involutional blepharoptosis according to age in a representative Korean population. We also determined the possible relationship between blepharoptosis and obesity by age group. Overall, the purpose of this study was to determine an appropriate age threshold for recommending evaluation for blepharoptosis among Korean adults.

## Methods

### Survey and subjects

This study used data from the Korean National Health and Nutrition Examination Survey (KNHANES-V), which was performed in 2010–2012 by the Korean Centers for Disease Control and Prevention and the Korean Ministry of Health and Welfare, Sejong, Republic of Korea. A total of 25,534 individuals in KNHANES-V were identified as candidates for this study. Participants aged less than 19 years (*n* = 5935) were excluded, as were adults with a previous history of eyelid or intraocular surgery, or with a medical condition that might affect the position of the eyelid or motility, including thyroid disease, systemic collagen disease, myopathy, cerebrovascular and cardiovascular disease (*n* = 1721). Non-operated adult patients with congenital blepharoptosis were included in this study. In total, 17,878 adults were included in the final analyses. All participants provided written consent forms prior to enrollment. The Institutional Review Board of the Korea Centers for Disease Control and Prevention (KCDC) reviewed and approved this nationally representative data study.

### Sociodemographic and lifestyle variables

Trained interviewers from the KNHANES-Vadministered standardized health examinations and questionnaires. Current smokers were defined as participants who currently smoked and had smoked more than 100 cigarettes in their lifetime. Participants were also categorized based on the quantity of alcohol consumed per day for the month prior to the interview; participants were considered heavy drinkers if they had consumed more than 30 g per day. Participants who performed moderate exercise at least five times per week for 30 min or more per session, or who performed vigorous exercise at least three times per week for 20 min or more per session, were considered regular exercisers. The educational level was classified as high if the respondent had completed a high school education. A monthly household income of less than 1092.40 United Sates dollars (USD) corresponded to the lowest income quartile.

### Anthropometric and biochemical measurements

Anthropometric measurements were performed by specially trained examiners. Body weight and height, measured with participants barefoot and wearing light clothing, were used to calculate the body mass index (BMI). Waist circumference (WC) was estimated to the nearest 0.1 cm in a horizontal plane at the midpoint from the iliac crest to the costal margin at the end of a normal expiration. Blood pressure was checked every 8 h via the right arm while the participant was in a sitting position, after about 5 min of relaxation, by a mercury sphygmomanometer (Baumanometer; Baum, Copiague, NY, USA). The definitive blood pressure was determined using mean values of the second and third measurements. A participant was classified as hypertensive if the systolic blood pressure was more than 140 mmHg or the diastolic blood pressure was more than 90 mmHg, or if the participant had been diagnosed with high blood pressure and was taking high blood pressure medication. The percent body fat (fat mass/total mass × 100, %) and sum of the lean soft tissue mass of the arms and legs were obtainedby dual-energy X-ray absorptiometry (DXA, QDR 4500 A; Hologic, Waltham, MA, USA) at mobile examination centers.

### Blepharoptosis definitions

The eyelid positions of all participants were examined by specially trained opticians who were resident physicians. The qualitycontrol of the ophthalmic survey was performed by the Epidemiologic Survey Committee of the Korean Ophthalmologic Society. Marginal reflex distance 1 (MRD1) was defined as the distance from the upper eyelid margin to the corneal light reflex in the primary position [13, 14]. The ophthalmic resident physicians with over 3 years of experience participated in this survey by directly measuring with a ruler. A differential diagnosis of blepharoptosis was made with particular attention paid to pseudoptosis associated with eyebrow ptosis and dermatochalasis. MRD1 values were obtained and sorted into five subclassifications: (1) ≥ 4.0 mm, (2) 3.0–3.9 mm, (3) 2.0–2.9 mm, (4) 1.0–1.9 mm, and (5) <  1.0 mm. Before data analyses, we defined blepharoptosis as an MRD1 of less than 2 mm for either eye. The levator function test (LFT) was also evaluated by measuring the upper eyelid excursion from downgaze to upgaze, excluding any influence of frontalis muscle function, and sorted into four subclassifications: (1) ≥ 12 mm, (2) 8–11 mm, (3) 5–7 mm, and (4) <  4 mm.

### Statistical analysis

Statistical analyses were conducted using the survey procedure of SAS Windows software (version 9.3; SAS Institute, Cary, NC, USA) to account for the complex sampling design from the KNHANES, which supplies nationally representative blepharoptosis prevalence estimated values. A two-sided *P* value less than 0.05 was considered statistically important. Fundamental characteristics of participants with and without blepharoptosis were expressed as proportions (% standard error, SE) for categorical variables and as means ± SE for continuous variables. The chi-square test or independent two-sample *t*-test was performed to compare differences in participant characteristics with or without the presence of blepharoptosis. The cutoff age in the prediction of blepharoptosis was defined as the point of largest statistical value by the Rao-Scott chi-square test.

## Results

### General characteristics of the study participants (Table 1)

The baseline characteristics of the study participants are presented in Table 1. The cross-sectional analyses included data on 17,878 adults (15,178 participants without blepharoptosis and 2160 participants with blepharoptosis). Among the eligible participants, the incidence of blepharoptosis was 12.08%. Table 1 shows the results of univariate analyses of the associations between blepharoptosis and demographic characteristics, lifestyle and medical factors, and obesity parameters. Based on univariate analyses, factors associated with the composite outcome of blepharoptosis were less drinking, less exercise, less income, less education, more metabolic syndromes, more hypertension, more severe diabetes, more cataracts, less employment, higher age, a higher BMI, and a greater WC. After adjusting for confounders, age, and obesity parameters including BMI and WC were closely related with blepharoptosis.

**Table 1:** The baseline characteristics of the studied individuals with or without blepharoptosis

	Blepharoptosis		P
	No (*n* = 15,718)	Yes (*n* = 2160)	
Smoke	23.8 (0.5)	22.1 (1.3)	0.238
Alcohol drinking	59.5 (0.6)	47.1 (1.5)	< 0.0001
Exercise	19.9 (0.5)	16.2 (1.3)	0.0069
Low income	14.7 (0.5)	33.4 (1.6)	< 0.0001
Education level (over high school)	74.2 (0.7)	33.8 (2.0)	< 0.0001
Metabolic syndrome	23.9 (0.5)	46.7 (1.4)	< 0.0001
Hypertension	24.8 (0.5)	51.2 (1.6)	< 0.0001
Diabetes	7.3 (0.3)	19.6 (1.1)	< 0.0001
Cataract	22.6 (0.7)	63.5 (2.0)	< 0.0001
Family history of ocular disease	20.9 (0.5)	14.1 (1.1)	< 0.0001
Occupation	65.4 (0.5)	53.5 (1.5)	< 0.0001
Age	43.9 ± 0.2	60.5 ± 0.7	< 0.0001
Body mass index	23.6 ± 0.04	24.3 ± 0.09	< 0.0001
Waist circumstance	80.7 ± 0.1	84.5 ± 0.3	< 0.0001

### Eyelid measurements and blepharoptosis prevalence according to age group (Table 2 and Fig. 1)

**Fig. 1:**
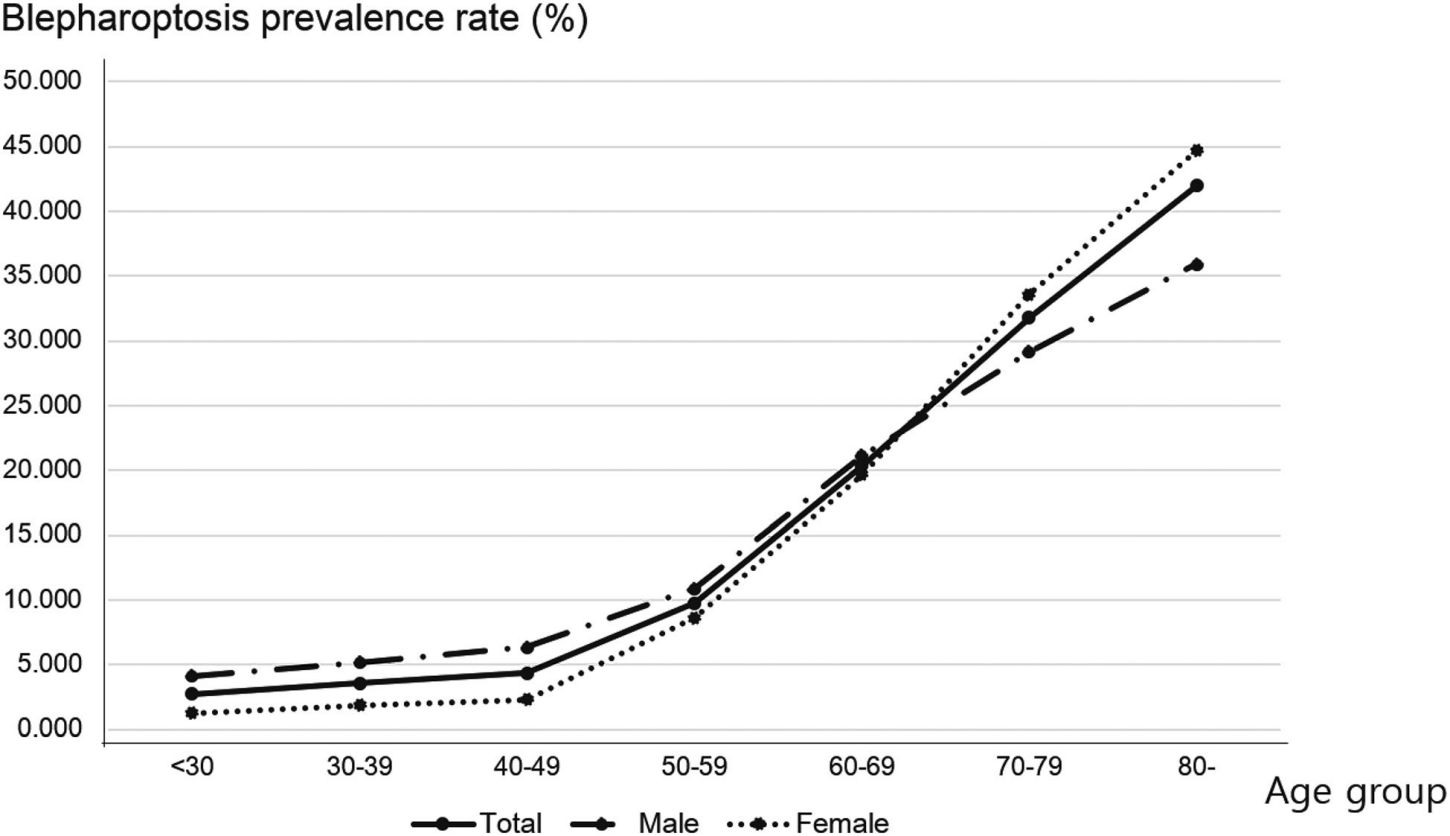
Prevalence of blepharoptosis among Korean males, females, and the total population according to age. The prevalence of blepharoptosis increased with age for all three groups

Table 2 shows the mean MRD1 (mm) and mean levator function test (LFT, mm) results for both eyes and for all age groups, as well as the blepharoptosis prevalence (%) according to age group. Both mean the MRD1 and mean LFT decreased, while blepharoptosis prevalence (%) increased, with increasing age. These relationships were especially apparent in the group over 60 years of age. Figure 1 shows the prevalence (%) of blepharoptosis for male, female, and all participants according to age group. Generally, blepharoptosis prevalence increased with increasing age, and increased more rapidly between the 50–59 and 60–69 years of age groups, and between the 60–69 and 70–79 years of age male, female, and total participants, because the slope of line was steeper in these sections compared to the other sections.

**Table 2:** Eyelid parameters including MRD1 and LFT according to age group

	Number	Age group						
		< 30	30–39	40–49	50–59	60–69	70–79	≥80
		2072	3248	3133	3421	3066	2399	539
Right MRD1% (SE^a^)	≥ 4 mm	52.36 (2.08)	52.13 (1.78)	46.45 (1.85)	32.76 (1.66)	21.69 (1.42)	11.86 (1.15)	7.65 (1.71)
	3–3.9 mm	32.13 (1.70)	33.97 (1.47)	34.13 (1.44)	38.13 (1.44)	33.74 (1.38)	28.50 (1.56)	23.15 (2.30)
	2–2.9 mm	12.97 (1.23)	10.92 (0.98)	15.65 (1.51)	20.56 (1.16)	25.67 (1.20)	31.03 (1.36)	30.40 (2.57)
	1–1.9 mm	2.08 (0.41)	2.53 (0.42)	3.10 (0.41)	7.05 (0.69)	13.87 (0.99)	20.47 (1.22)	23.11 (2.33)
	< 1 mm	0.46 (0.16)	0.46 (0.15)	0.68 (0.20)	1.50 (0.27)	5.03 (0.64)	8.15 (0.82)	15.69 (2.31)
Left MRD1% (SE)	≥ 4 mm	52.38 (2.06)	52.21 (1.77)	46.68 (1.86)	32.92 (1.67)	21.49 (1.43)	11.89 (1.16)	7.35 (1.70)
	3–3.9 mm	31.69 (1.68)	33.39 (1.47)	34.01 (1.42)	37.92 (1.39)	33.78 (1.45)	28.95 (1.60)	22.83 (2.32)
	2–2.9 mm	13.26 (1.27)	11.05 (0.98)	15.34 (1.51)	20.37 (1.16)	20.37 (1.21)	29.95 (1.35)	29.76 (2.48)
	1–1.9 mm	2.33 (0.44)	2.86 (0.43)	3.31 (0.44)	7.48 (0.66)	13.25 (1.00)	21.13 (1.29)	24.15 (2.33)
	< 1 mm	0.35 (0.14)	0.49 (0.15)	0.58 (0.17)	1.30 (0.26)	5.19 (0.67)	8.08 (0.86)	15.93 (2.06)
Rigth LFT % (SE)	≥ 12 mm	81.47 (1.48)	81.13 (1.44)	74.31 (1.62)	63.51 (1.59)	47.73 (1.73)	32.50 (1.84)	23.67 (2.62)
	8–11 mm	17.86 (1.45)	17.97 (1.36)	24.51 (1.58)	33.70 (1.52)	45.43 (1.61)	54.07 (1.81)	55.22 (2.68)
	5–7 mm	0.67 (0.22)	0.89 (0.34)	0.95 (0.23)	2.52 (0.41)	5.68 (0.67)	11.84 (1.03)	16.91 (2.13)
	< 4 mm	–	0.02 (0.02)	0.23 (0.15)	0.28 (0.15)	1.17 (0.47)	1.60 (0.49)	4.20 (1.73)
Left LFT % (SE)	≥ 12 mm	81.91 (1.46)	81.75 (1.43)	74.92 (1.60)	64.55 (1.55)	47.90 (1.75)	32.68 (1.84)	23.48 (2.66)
	8–11 mm	17.35 (1.42)	17.30 (1.36)	23.74 (1.56)	32.29 (1.45)	45.58 (1.61)	54.00 (1.73)	56.10 (2.74)
	5–7 mm	0.74 (0.27)	0.93 (0.34)	1.11 (0.26)	2.87 (0.44)	5.38 (0.67)	11.89 (1.06)	15.74 (2.08)
	< 4 mm	–	0.02 (0.02)	0.23 (0.11)	0.28 (0.15)	1.14 (0.47)	1.44 (0.48)	4.68 (1.77)
Blepharoptosis (%)		2.76	3.57	4.35	9.75	20.36	31.77	41.99

^a^SE means standard error

### Blepharoptosis prevalence according to age group and obesity parameters (Figs. 2 and 3)

**Fig. 2:**
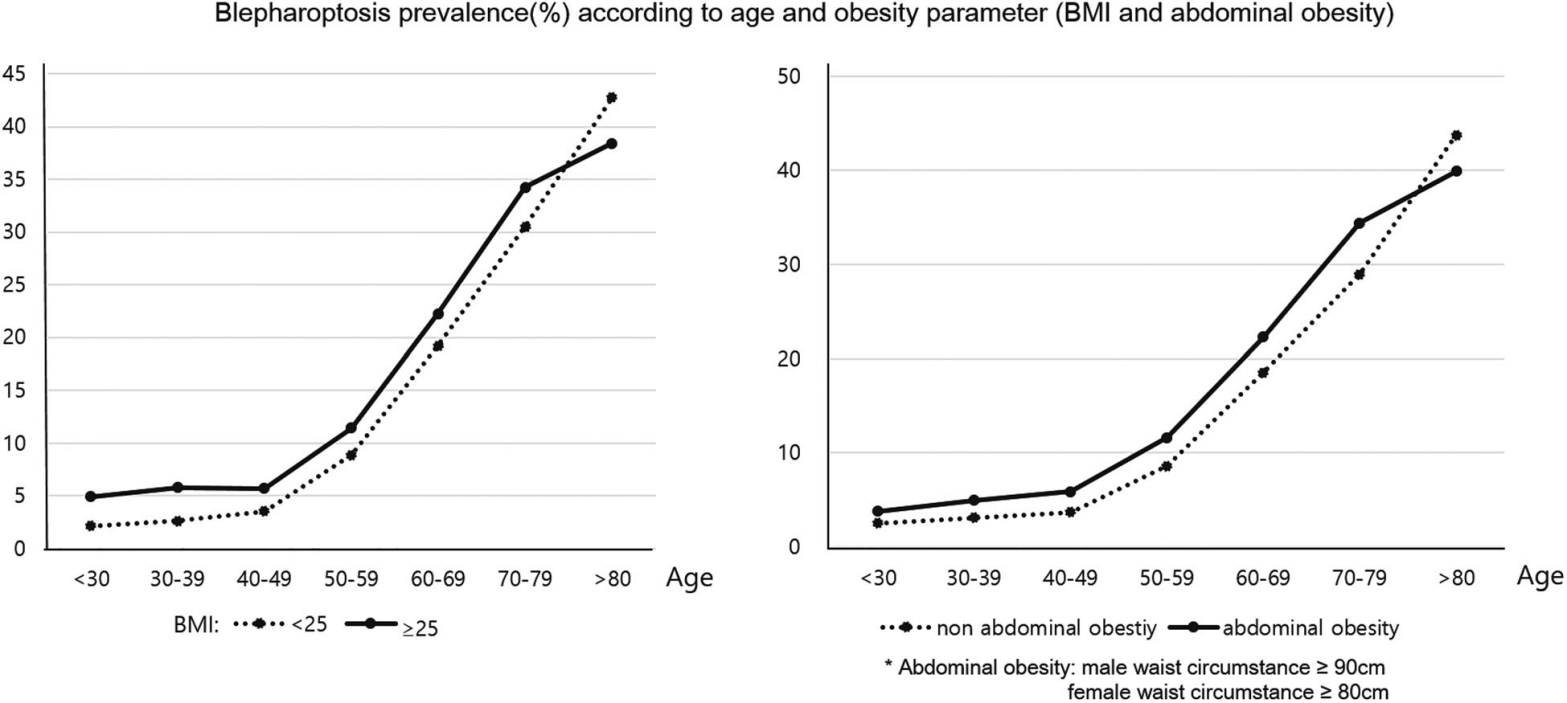
Blepharoptosis prevalence (%) according to age and obesity parameters [body mass index (BMI) and abdominal obesity (waist circumference, WC)]. The prevalence of blepharoptosis increased with a higher BMI (**a**) and larger WC (**b**)

**Fig. 3:**
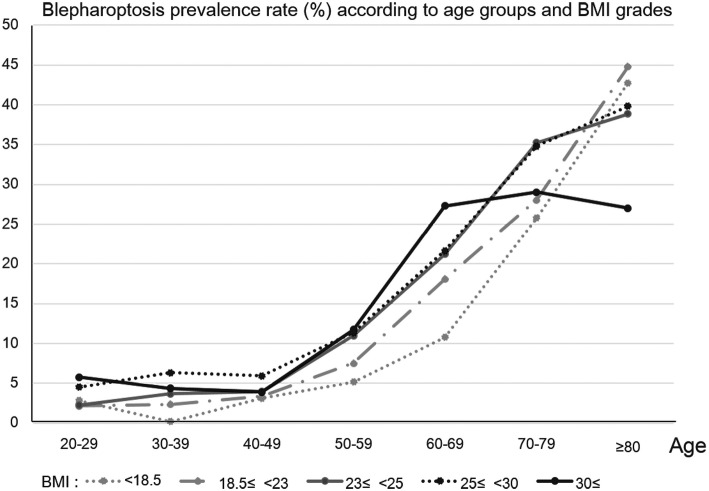
Blepharoptosis prevalence (%) according to age group and BMI. In general, the prevalence of blepharoptosis increased with higher BMI, which were more prominent between the 50–59 and 60–69 years of age groups, and between the 60–69 and 70–79 years of age groups. However, participants aged more than 70 years and with a BMI more than and equal to 30 were less likely to have blepharoptosis

Figure 2 shows blepharoptosis prevalence (%) according to age and obesity parameters, including BMI and abdominal obesity (WC). The blepharoptosis prevalence was higher among participants with a BMI more than and equal to 25 versus those with a BMI less than 25. Blepharoptosis prevalence was also higher in the participants with a WC more than and equal to 90 cm (males) and more than equal to 80 cm (females) versus those with a WC less than 90 cm (males) and less than 80 cm (females). Figure 3 shows the association between blepharoptosis and BMI according to age group. In the 50–59 and 60–69 years age groups, blepharoptosis incidence was higher, and the participants had higher BMI scores. The association between blepharoptosis incidence and obesity severity in these groups was stronger than that in the other groups. However, in the 70–79 and 80–89 years age groups, extremely obese participants (BMI more than 30) showed a sudden decrease in the prevalence of blepharoptosis.

### Optimal cutoff age for recommending blepharoptosis evaluation (Fig. 4)

**Fig. 4:**
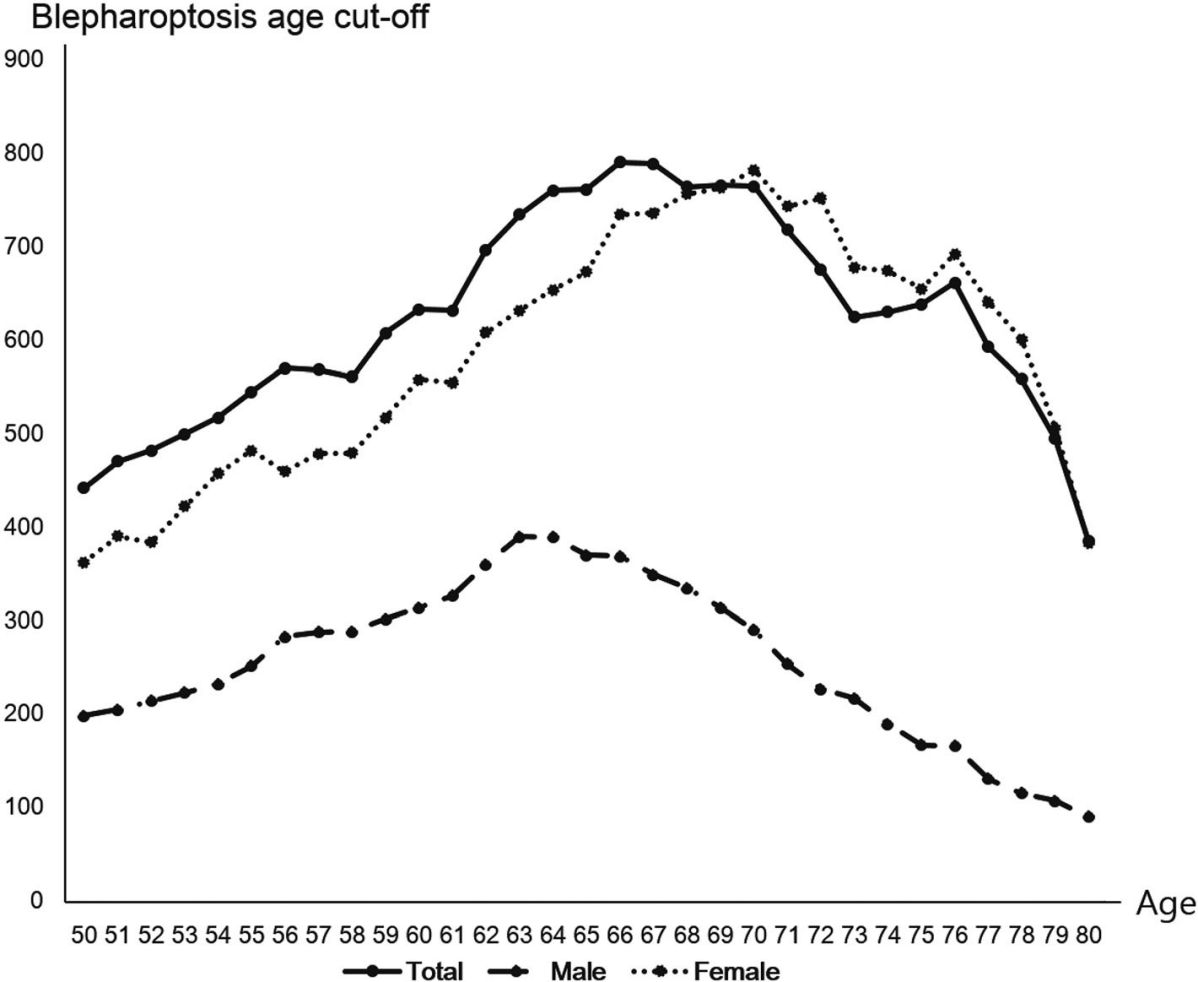
The cutoff age for recommending blepharoptosis evaluation was 66 years in the total population, 63 years in males, and 71 years in females. The cutoff age used in the prediction of blepharoptosis was defined as the point of largest statistical values using the Rao-Scott chi-square test

Finally, we investigated the cutoff age of for recommending blepharoptosis evaluation. The cutoff age in the prediction of blepharoptosis was defined as the point of largest statistical values using the Rao-Scott chi-square test. Figure 4 shows that the optimal cutoff age was 66 years for all participants, 63 years for males, and 71 years for females.

## Discussion

This study suggested that among the general Korean population, evaluation for blepharoptosis was recommended for the total population, males, and females at 66 years, or at 63 and 70 years for males and females, separately. The estimated prevalence of blepharoptosis in our study was 12.08%. This result is consistent with previous reports. Among Korean adults aged more than 40 years, the prevalence rate of age related blepharoptosis was 13.5% [15]. An estimated 11.5% of adults in the UK aged more than 55 years had blepharoptosis [16]. Thapa et al. reported in a hospital-based, cross-sectional study conducted in Nepal that 10.7% of ptosis cases were of aponeurotic ptosis [17].

Moderate to severe blepharoptosis can impair visual function (e.g., loss of superior visual fields) [6] cause psychological distress (e.g., anxiety and depression) [5], reduce health-related quality of life [18], and cause tension-related headaches [7–9]. Blepharoptosis in older adults may be underestimated or ignored because both physicians and patients consider the condition to be a result of the aging process. However, surgical repair of involutional blepharoptosis is not a difficult process, and most cases can be easily repaired under local anesthesia within a few hours [12]. Health-related quality of life outcomes are becoming increasingly important in determining the allocation of healthcare resources, and identifying these outcomes allows clinicians to better inform patients. Determination of an age cutoff for blepharoptosis evaluation is therefore important for patients and physicians considering the surgical benefits of blepharoptosis repair surgery.

In our study, the prevalence of blepharoptosis increased with age. MRD1 values and LFT scores decreased with age, while the prevalence of blepharoptosis increased. This can be explained by aponeurotic or periorbital changes. Aponeurotic dehiscence or disinsertion is a well-known cause of involutional ptosis [19]. Guyuron and Harvey reported that blepharoptosis and enophthalmos can be caused by periorbital and orbital aging, and there was a strong association between age-related enophthalmos and upper eyelid ptosis, which was undetected [11]. Aponeurotic abnormalities accompanied by orbital and periorbital aging-related changes can therefore cause involutional blepharoptosis, with age itself being one of strongest risk factors for involutional blepharoptosis.

Figures 1 and 2 show that, in this study, more obese participants were more likely to have blepharoptosis. Similarly, Paik et al. reported that obesity parameters (BMI, WC, and percent body fat) were strongly associated with age-related blepharoptosis [20]. However, when comparing blepharoptosis prevalence according to age group and BMI, participants aged more than 70 years and with a BMI more than and equal to 30 were less likely to have blepharoptosis. This might have been due to the exclusion of extremely obese adults aged more than 70 years from the study, on the assumption that they would had other severe medical disorders, or to the survival rate of extremely obese adults aged more than 70 years being lower than that of the younger participants to their higher risk of severe cardiovascular or cerebrovascular disorders [21, 22].

There were some limitations to this study. First, because it used a cross-sectional design, disease causality could not be established from the investigated associations. Second, the study included a nationally representative population of Korean males and females, so our findings may not be generalizable to other populations or racial groups. Finally, because the most important variable, MRD1, was conducted by several inspectors instead of one expert due to the high number of participants, there could be biases among inspectors. In spite of these limitations, this is the first population-based study to measure eyelid parameters in detail, and to determine the cutoff age of age-related involutional blepharoptosis.

In conclusion, this was the largest population-based study to determine the cutoff age for recommending blepharoptosis evaluation from representative data on involutional blepharoptosis among Korean adults. We suggest that for the general population, evaluation for blepharoptosis should be done at 66 years of age, or at 63 and 70 years for males and females, separately. In summary, this study provided appropriate age thresholds for involutional blepharoptosis among the general population of Korea. The results also suggest that past a certain age, Koreans should undergo regular blepharoptosis evaluations.

## Conclusions

There was astrong association between older age and the prevalence of blepharoptosis. Moderate to severe blepharoptosis resulted in poor visual function and exacerbated headaches and depression, leading to decreased quality of life. This study could suggest that for the general Korean population, evaluation for blepharoptosis should be done at 66 years of age, or at 63 and 70 years of age for males and females, separately.

## Data Availability

All data generated and analyzed during this study are included in relevant published articles.

## Abbreviations

% SE: % Standard error
BMI: Body mass index
KNHANES: The Korean National Health and Nutrition Examination Survey
LFT: Levator function test
MRD1: Marginal reflex distance 1
SE: Standard error
USD: United States Dollars
WC: Waist circumference
